# Enhancing vocational English learning through peer tutoring

**DOI:** 10.1186/2193-1801-3-S1-O7

**Published:** 2014-12-04

**Authors:** Miranda Man-wai Lam, Avery, Chung-woon Chan

**Affiliations:** Language Centre, Youth College (Kowloon Bay), Kragujevac, Hong Kong

## Background

This study aimed to explore how the pilot Peer Tutoring Programme in Youth College (Kowloon Bay) enhanced students’ vocational English learning. The findings showed that the program had positive effects on the students’ academic performance, increased their learning motivation, boosted their confidence and encouraged active learning outside the classroom.

According to Topping [1], peer tutoring, which involves students helping their peers to learn and, at the same time, helping themselves to learn through teaching, is one of ‘the longest established and most intensively researched forms of peer learning’. If organized appropriately, it clearly benefits students academically, cognitively and socially. This approach had been tried out by a few language teachers in some of the Vocational English classes in Youth College (Kowloon Bay) since 2009.

## Methods

Nineteen students of The Diploma in Vocational Education (DVE) (S.3 Progressing & S.6 In-take) were chosen as peer tutors (PTs) in the academic year 2009-10. They were responsible for facilitating their peers’ understanding of the lesson content in class and answering their peer tutees’ questions. In the academic year 2010-11, 67 DVE students were selected as PTs to help in 13 Vocational English classes. This mode of learning received overwhelming positive feedback from teachers, PTs and peer tutees. Thus, to determine the extent of its effectiveness in enhancing students’ vocational English learning, a more structured pilot Peer Tutoring Programme was launched in the academic year 2013-14. Thirty-seven DVE students were nominated to serve as PTs, and each was assigned two to three peer tutees. The PTs attended several sessions of training on how to effectively communicate with their peer tutees and better guide them in learning English. Also, an introductory workshop was conducted at the beginning of the program to ensure that PTs understood their role in this project. Lesson observations and interviews were conducted with teachers and students involved.

## Results

The program had positive effects on students’ academic performance. Nearly every PT and tutored student achieved the learning outcomes of the Vocational English modules. For the PTs, as suggested by Gaustad [2], the time spent on revising with their peer tutees and explaining the learning materials to them reinforced their understanding of the subject knowledge. As for the peer tutees, the PTs helped them to study more effectively by giving them individual instruction and support whenever they encountered problems. The students’ learning motivation was increased, which was in line with previous findings [3]. The PTs reported being eager to study the learning materials before classes and to consult their teachers frequently to be better prepared for teaching their peer tutees. Meanwhile, peer tutees were willing to revise what they had learnt and continually seek support from their PTs and teachers. They also expressed more positive attitudes towards English learning. The scheme also boosted students’ confidence in learning English as they had support from their peers. Furthermore, the program fostered students’ ownership in learning and encouraged outside classroom learning. Some PTs voluntarily studied with their peer tutees on campus after school. A bigger pleasant surprise was that a few PTs organized an online video discussion group to practice vocabulary pronunciation and oral presentation with their peer tutees. All of these additional experiences enabled PTs to feel worthwhile and valuable, while giving peer tutees more opportunities to learn from their peers outside normal lessons.

## Conclusions

The program was effective in helping students academically and socially. Further studies on how structured peer-tutoring programs can enhance English learning are highly recommended. It is also worth exploring ways to extend the scale of the program to benefit more students.
